# BCYRN1, a c-MYC-activated long non-coding RNA, regulates cell metastasis of non-small-cell lung cancer

**DOI:** 10.1186/s12935-015-0183-3

**Published:** 2015-04-01

**Authors:** Tao Hu, Yu-Run Lu

**Affiliations:** Sichuan Provincial People’s Hospital, No. 32, Section 2, 1st Ring Road (West), Chengdu City, 610072 China

**Keywords:** BCYRN1 (brain cytoplasmic RNA 1), lncRNA (long non-coding RNA), c-MYC, Metastasis, NSCLC (non-small-cell lung cancer)

## Abstract

**Background:**

Long non-coding RNAs (lncRNAs) are increasingly implicated in the regulation of the progression of malignancy.

**Aim:**

To clarify the relations among BCYRN1 (brain cytoplasmic RNA 1, a long non-coding RNA), c-MYC and cell metastasis of non-small-cell lung cancer (NSCLC).

**Methods:**

Real-time PCR was used to measure expression of BCYRN1 in NSCLC. Knockdown and overexpression of c-MYC were respectively performed using shRNA and lentivirus to investigate its effect on BCYRN1 expression. BCYRN1 was respectively knockdown and overexpressed by siRNA and BCYRN1 mimics to investigate its role in regulating cell metastasis *in vitro*. ChIP (chromatin immunoprecipitation) assay was performed to confirm the binding of c-MYC to the promoter of BCYRN1. Expression levels of matrix metalloproteinases (MMP9 and MMP13) were determined using real-time PCR and Western blotting.

**Results:**

BCYRN1 is upregulated and targeted by c-MYC in NSCLC, leading to the increase of cell motility and invasiveness. RNA interference and lentivirus infection showed a positive correlation between the expressions of c-MYC and BCYRN1. ChIP assay confirmed the binding of c-MYC to the promoter region of BCYRN1 gene. *In-vitro* cell metastasis experiments demonstrated that BCYRN1 was necessary in the c-MYC-regulated cell migration and invasion. The mRNA and protein expression levels of MMP9 and MMP13 descended with the decreasing BCYRN1 level and ascended with the upregulation of BCYRN1.

**Conclusion:**

These findings uncover a regulatory mechanism in NSCLC cells involving the metastasis-promoting lncRNA BCYRN1 that improves expressions of the key metastasis-supporting proteins MMP9 and MMP13.

## Introduction

Lung cancer is determined very largely by tobacco smoking, and had the highest incidence in men and the third in women [1]. With a 5-year overall survival of 10–15%, lung cancer account for more than one-quarter of all cancer deaths, which is the highest mortality [1,2]. Non-small-cell lung cancer (NSCLC), including adenocarcinoma, squamous cell carcinoma, and large cell carcinoma, composes 80–85% of lung cancers, and almost half of patients with newly diagnosed NSCLC have metastatic disease [3]. It is precisely owing to the high rate of metastasis that NSCLC have become the most lethal human cancer [4].

Long non-coding RNAs (lncRNAs) refer to the non-coding RNAs that were not shorter than 200 nt [5]. It has been found more than once that lncRNA expressions are involved in cancer metastasis [6-8], for example, an lncRNA named MALAT-1 is proved to induce cell migration and tumor metastasis of NSCLC through influencing the expression of motility-related genes [9,10]. In accordance to the literature, BCYRN1 (brain cytoplasmic RNA 1) a 200-nucleotide lncRNA, is found highly expressed in some carcinomas of the breast, cervix, oesophagus, lung, ovary, etc., but normally not detectable in the corresponding normal tissues [11].

Studies have indicated its possible role in progression of carcinoma via translational repression of yet to be discovered metastasis suppressors [12,13]. As an oncogene, *c-MYC* is closely correlated with metastasis of NSCLC [4], and there is (are) a binding site(s) for c-MYC in BCYRN1 promoter area, based on the online search results (http://www.sabiosciences.com/chipqpcrsearch.php?gene=BCYRN1&species_id=0&factor=c-Myc&ninfo=n&ngene=n&nfactor=n; http://liweilab.genetics.ac.cn/tm/gene.php?st=gn&gn=c-Myc-binding%20protein&gi=618&ti=9606). During tumor metastasis, extracellular matrix (ECM) interacted with tumor cells in various ways [14]. MMPs (matrix metalloproteinases), a family of more than 20 secreted or transmembrane proteins, have the ability to digest ECM and basement membrane components, which has been shown to have correlations with the metastatic potential of tumor cells [15].

However, the role of BCYRN1, and its correlation with c-MYC and MMPs in metastasis of lung carcinoma are not well understood. The present study was done to preliminarily exposit a possible role of BCYRN1 in regulating cell metastasis of NSCLC.

## Materials and methods

### Tissues

Tumor tissue and the adjacent non-tumor tissue samples were obtained from 20 patients who were diagnosed as NSCLC. The research was performed under the guidance of the institutional ethical guidelines, and the use of human lung tissues was approved by the Medical Ethics Committee of Sichuan Provincial People’s Hospital. Every patient involved in the study has signed the written informed consent. The adjacent non-tumor tissue samples were used as controls. All samples were snap-frozen and stored in liquid nitrogen for further use.

### Cell lines

Five human NSCLC cells lines, including A549, H1299, SPCA-1, H520 and L78, and a human normal cell line 16HBE (human bronchial epithelial cells) were purchased from Cell Bank of Type Culture Collection of Chinese Academy of Sciences (Shanghai, China). A549, H1299 and 16HBE cells were cultured in DMEM medium (90%) with 10% fetal bovine serum (Hyclone, USA), and the rest cell lines were cultured in RPMI-1640 medium supplemented with 10% fetal bovine serum (Hyclone, USA) in a humidified atmosphere of 5% CO_2_ at 37°C.

### RNA isolation and real-time polymerase chain reaction (PCR)

Total RNA was prepared from lung tissues or cells using Trizol reagent (Invitrogen, CA, USA) and cleaned up using RNeasy Mini Kit (Qiagen, Hilden, Germany). BCYRN1 expression level was determined on an ABI 7900HT system using SYBR green qPCR assay (Takara, Dalian, China). Primer sequences were as follows: BCYRN1, forward primer: 5′-CTGGGCAATATAGCGAGAC-3′, reverse primer: 5′-TGCTTTGAGGGAAGTTACG-3′; c-MYC, forward primer: 5′-TCAAGAGGCGAACACACAAC-3′; reverse primer: 5′-GGCCTTTTCATTGTTTTCCA-3′; GAPDH (used for normalization), forward primer: 5′-ACCACAGTCCATGCCATCAC-3′, reverse primer: 5′-TCCACCACCCTGTTGCTGTA-3′. The condition of real-time PCR was 95°C for 2 min, followed by 40 cycles of 15 s at 95°C and 1 min at 55°C. The data were analyzed using 2^-ΔΔCt^ method.

### Small interfering RNA (siRNA) and short hairpin RNA (shRNA)

Control and BCYRN1-specific siRNAs were synthesized by Shanghai GenePharma Co., Ltd (Shanghai, China) and used for transfection into A549 cells according to the manufacturer’s protocols. As described by Zhuang et al. [16], lentiviral vector pLKO-1 (Sigma, St Louis, MO, USA) containing *c-MYC*-specific shRNA (shRNA-*c-MYC*) was used for the inhibition of c-MYC expression. The *c-MYC*-targeting sequence was composed of the nucleotides from 1567 to 1585 in the previously reported sequence (GenBank gene accession No. NM_002467.3). The lentiviral vector that contained an irrelevant sequence was used as a negative control. The rest steps were referring to the previous literatures [16-18]. The lentivirus-infected A549 cells were monitored through the observation of green fluorescent protein expression. The knockdown of c-MYC expression in the cells was confirmed by Western blotting.

### Overexpression of c-MYC in A549 cells

A549 cells were infected with lentiviral plasmid pWPXL-*c-MYC* (Addgene, plasmid 36980, Lausanne, Switzerland) using Lipofectamine 2000 (Invitrogen, CA, USA). An empty plasmid pWPXL served as a negative control.

### Chromatin immunoprecipitation (ChIP)

ChIP assay was carried out using an EZ-Magna ChIP kit (Millipore, Billerica, MA, USA) according to the manufacturer’s protocol. Briefly, A549 cells (5 × 10^6^) were treated with 1% formaldehyde for 10 min for crosslinking, and then quenched by the addition of 0.125 M glycine. The cells were scraped with PBS (phosphate buffer saline) solution and gathered after centrifugation at 800 g for 5 min at 4°C. Then, the cross-linked cells were resuspended in 1 % SDS (sodium dodecyl sulfate) lysis buffer and the soluble chromatin was sheared to fragment DNA of about 400 bp in length by nuclear lysis buffer. The fragmented chromatin samples were aliquoted as genomic input DNA or immunoprecipitated with 1 g c-MYC antibodies or rabbit IgG, incubated at 4°C with rotation for 4 h. Immunocomplexes, collected by magnetic separator, were washed and eluted with 1% SDS and 0.1 M NaHCO_3_. DNA was purified on spin columns. The ChIP products and genomic input DNA were quantitatively analyzed by real-time PCR (primer sequences are as follows: forward primer: 5′-ATGTTGCTCAGGGAGGTCTC-3′; reverse primer: 5′-GGCTTCTGTCCCTACACCAT-3′). ChIP data were presented as percentage of input normalized to control purifications.

### Cell migration and invasion assays

For the migration assay, referring to the methods described in the report by Jiang *et al*. [19], 5 × 10^4^ cells were injected into each well of the 24-well transwell chambers with 8.0-μm–pore polycarbonate filter inserts (Costar, San Diego, CA, USA). For the invasion assay, 5 × 10^4^ cells were placed into the upper chamber with an insert coated with Matrigel (BD Bioscience, Woburn, MA, USA). Complete media were added to the lower chamber. After incubation for several hours, the cells remaining on the upper membrane were scraped with cotton wool, and the cells that had migrated or invaded to the other side of the membrane were stained with 0.3% crystal violet. Cell migration or invasion was evaluated by counting whole cell numbers at single filter under an IX71 inverted microscope (Olympus, Tokyo, Japan) at 100× magnification. All experiments were independently repeated three times.

### Western blotting

After the A549 cells were treated with c-MYC inhibitor 10058-F4, or infected with pWPXL-*c-MYC* or pWPXL-NC, or transfected with siRNA-NC, siRNA-*BCYRN1*-1 or siRNA-*BCYRN1*-2, the medium was replaced with serum-free medium. The cells were incubated for another 15 h, following which, the conditioned medium was collected and total proteins from it for c-MYC was electrophoresed on SDS-polyacrylamide gel. C-MYC was then transferred to polyvinylidene fluoride (PVDF) membrane and blocked with nonfat, dry milk and 0.1% Tween 20 in TBS. The membranes were probed overnight with primary antibodies against c-MYC (Santa Cruz Biotechnology, Santa Cruz, CA). The membranes were then developed with peroxidase-conjugated secondary antibodies (Wuhan Boster Biological Technology, Ltd., Wuhan, China) for 1 h and enhanced chemiluminescence reagents (Beyotime, Shanghai, China). Protein GAPDH served as a control to equal protein loading.

### Enzyme-linked immunoadsorbent assay (ELISA) for MMP9 and MMP13

The concentrations of MMP9 and MMP13 in the supernatant of the media were determined using commercially available ELISA kits (Invitrogen, CA, USA). The MMP9 and MMP13 present in the samples or standards were bound in wells that had been pre-coated with the antibody. This phase lasted 1 h for MMP9 and 2 h for MMP13. A second antibody labeled with peroxidase was added for antigen-antibody immunoreaction in the second incubation phase (2 h for MMP9 and 1 h for MMP13). The amount of peroxidase that binds to each well was determined by the addition of a pre-prepared medium (tetramethylbenzidene, TMB). The reaction was blocked by adding a 1 M sulfuric acid, and the absorbance of the solution was measured at 450 nm using a microplate reader (BIO-RAD iMARK 680, Laboratories Inc., Hercules, CA, USA) within 10 min of the last phase of this experiment. Concentrations of MMP-9 and MMP-13 in the samples were determined by extrapolation from an adapted standard curve. All assays were performed in triplicate [20].

### Statistical analysis

All values were processed with a software DPS v9.50, and expressed as mean ± standard deviation. Student’s t test was used to analyze the difference between groups. The results were considered to be statistically significant, if *p* < 0.05.

## Results

### Upregulation of BCYRN1 and c-MYC expressions in NSCLC

**Figure 1:**
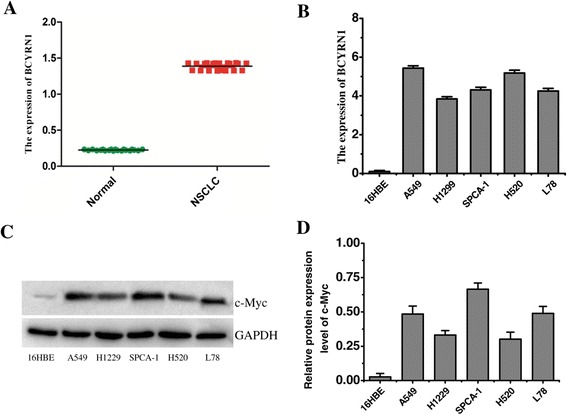
**BCYRN1 expression level in NSCLC tissue (A) and cell lines (B), and c-MYC protein level in NSCLC cell lines (C, D).** Normal: the adjacent normal tissue, used as a control; 16HBE: a normal human bronchial epithelial cell line.

The preliminary experiment demonstrated that the expression of lncRNA BCYRN1 in NSCLC tissue was significantly (*p* = 0.003, n = 20) higher than that in the adjacent normal tissue (Figure 1 A). Likewise, BCYRN1 in each selected NSCLC cell line, including A549, H1299, SPCA-1, H520 and L78, also showed a higher expression level than that in normal human bronchial epithelial cells (16HBE) (Figure 1 B). Western blotting analysis indicated that c-MYC level in all of these NSCLC cell lines are significantly higher than that in 16HBE cells (Figure 1 C, D).

### High expression of BCYRN1 originated from c-MYC’s binding to its promoter

**Figure 2:**
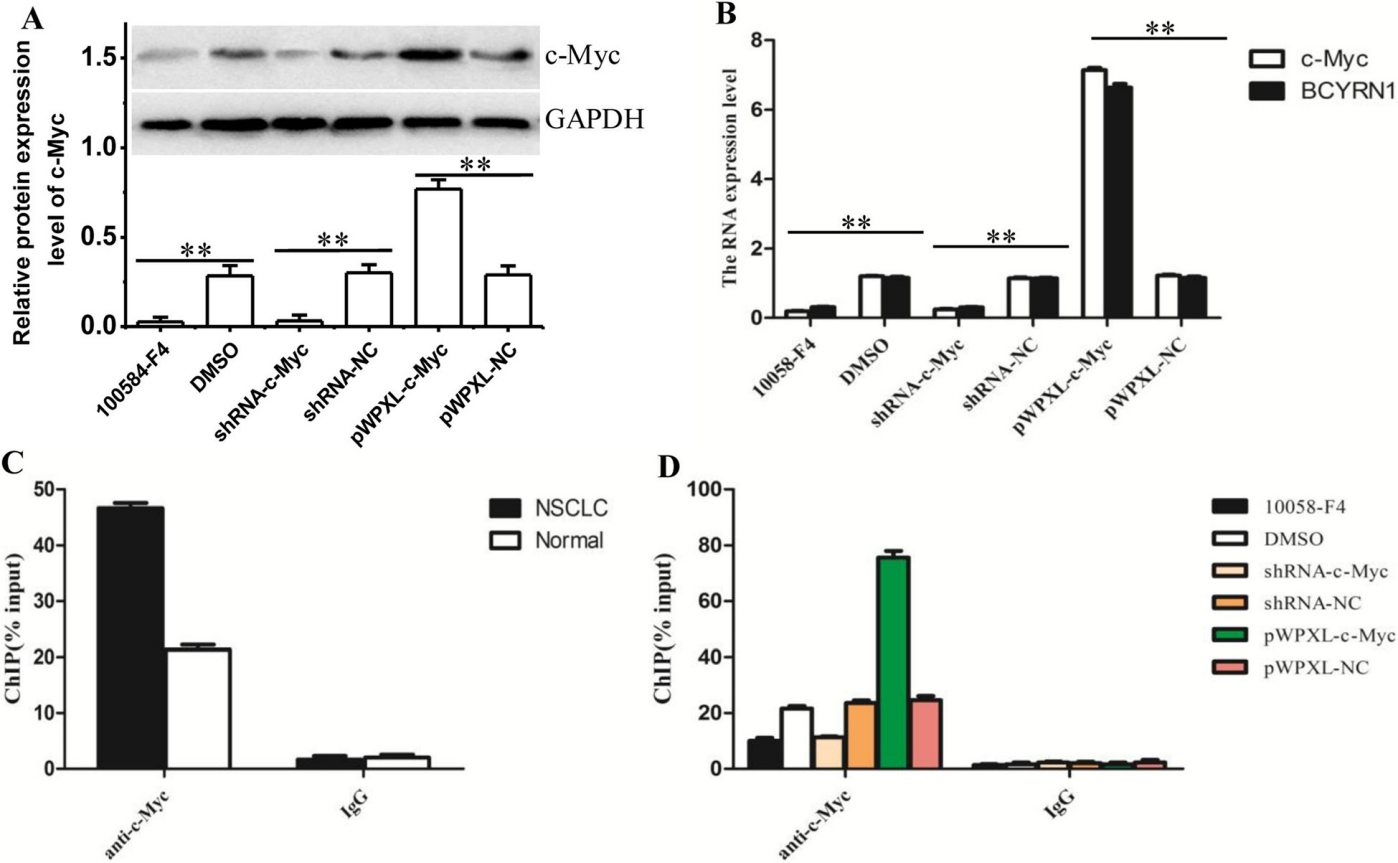
**Protein c-MYC binds to the promoter of BCYRN1.** A/B. Protein **(A)** and mRNA **(B)** levels of BCYRN1 and c-MYC in the NSCLC cells infected with pWPXL. 10058-F4: a small-molecule c-MYC inhibitor; groups shRNA-c-MYC, shRNA-NC (NC: negative control), pWPXL-c-MYC and pWPXL-NC respectively represented the groups where NSCLC cells were transfected with specific shRNA against c-MYC, non-specific shRNA, and infected with pWPXL-c-MYC vector or the pWPXL vector containing non-specific sequence. **(C)** ChIP assay showed binding of c-Myc to the BCYRN1 promoter in NSCLC tissues. **(D)** ChIP assay showed binding of c-Myc to the BCYRN1 promoter in A549 cells. Soluble chromatin of A549 cells and NSCLC tissue were immunopurified using anti-c-MYC antibody and quantitative real-time PCR was used to measure the level of enrichment on BCYRN1. ** *p* < 0.01, indicated a very significant difference, compared with negative control.

As mentioned above in the **Introduction**, in the promoter region of BCYRN1, a binding site for c-MYC lies. Therefore, various treatments, including the inhibition of c-MYC by c-MYC inhibitor or c-MYC knockdown and c-MYC overexpression, were used to investigate the relation between BCYRN1 and c-MYC. After the treatment of A549 cells with the small-molecule c-MYC inhibitor 10058-F4, the RNA expressions of c-MYC and BCYRN1 both markedly descended (**p < 0.01), compared with the group only treated with DMSO (dimethylsulfoxide) (Figure 2 A, B). When c-MYC was knockdown by its specific shRNA, BCYRN1 expression was also reduced (**p < 0.01, shRNA-*c-MYC* VS shRNA-NC) (Figure 2 A, B). Furthermore, after the A549 cells were infected with pWPXL-*c-MYC* vector, BCYRN1 overexpressed with the overexpression of c-MYC, and both of their levels were significantly higher than those in the cells infected with pWPXL-NC (**p < 0.01) (Figure 2 A, B).

According the results of ChIP assay, the enrichment levels of c-MYC in A549 cells (Figure 2 D) and NSCLC tissues (Figure 2 C) were astonishingly consistent with the above results. These results proved that high expression of BCYRN1 in NSCLC cells originated from c-MYC’s binding to the promoter.

### Migration and invasion of A549 cells were regulated by c-MYC-activated BCYRN1

**Figure 3:**
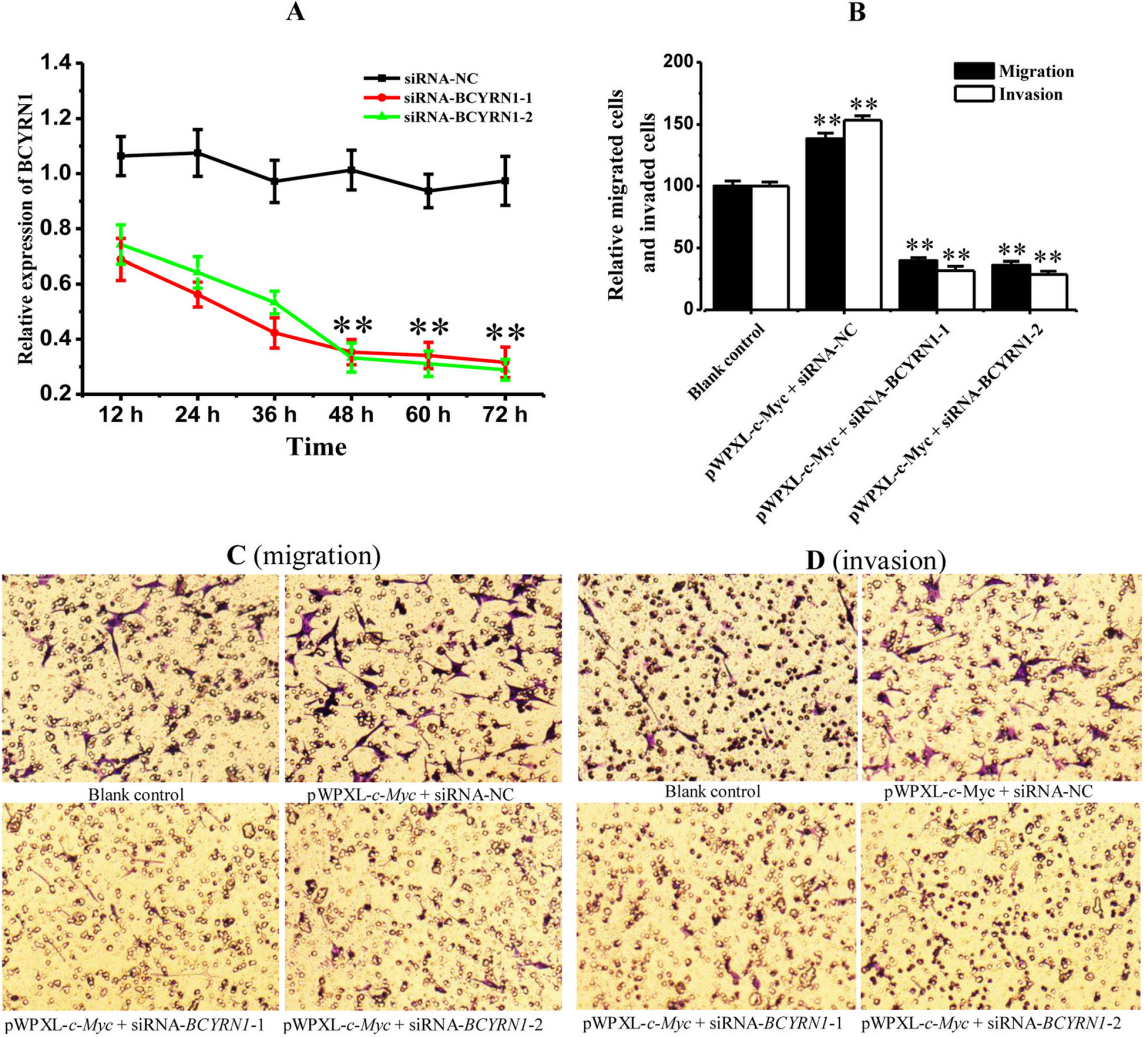
**The change of BCYRN1 level in A549 cells (A), and migration and invasion of A549 cells (B ~ D; B: the quantitative results for figures C and D) after siRNA interference in c-MYC overexpressed cells.** *Blank control*: the cells alone infected with pWPXL*-c-Myc*. pWPXL*-c-Myc +* siRNA-NC: the cells jointly treated with lentivirus (pWPXL-*c-Myc*) infection and siRNA transfection together. pWPXL*-c-Myc +* siRNA-*BCYRN1*-1 or −2: the cells jointly treated with lentivirus (pWPXL-*c-Myc*) infection and siRNA transfection (the two specific siRNAs of BCYRN1were respectively named as siRNA-*BCYRN1*-1 and siRNA-*BCYRN1*-2). Magnification: 100 ×. ** *p* < 0.01, indicated a very significant difference, compared with control.

Real-time PCR analysis revealed that the interference efficiency of the siRNA against BCYRN1 at 48 h after transfection was 65.15% and 67.13%, respectively (Figure 3 A). As shown in Figure 3 (B ~ D), when treated with pWPXL-*c-MYC* and siRNA-NC, namely, c-MYC-BCYRN1 combination was promoted, both the numbers of migrated cells and invaded cells were significantly larger than those of the untreated cells (**p < 0.01). While the A549 cells which were treated with pWPXL-*c-MYC* and BCYRN1 specific siRNAs simultaneously, meaning the combination of c-MYC and BCYRN1 were downregulated, showed various less migrated and invaded cells than the control (**p < 0.01, Figure 3 B). All of the above results suggested that migration and invasion of A549 cells were regulated by c-MYC-activated BCYRN1.

### MMP 9 and MMP 13 were involved in BCYRN1-mediated cell migration and invasion

**Figure 4:**
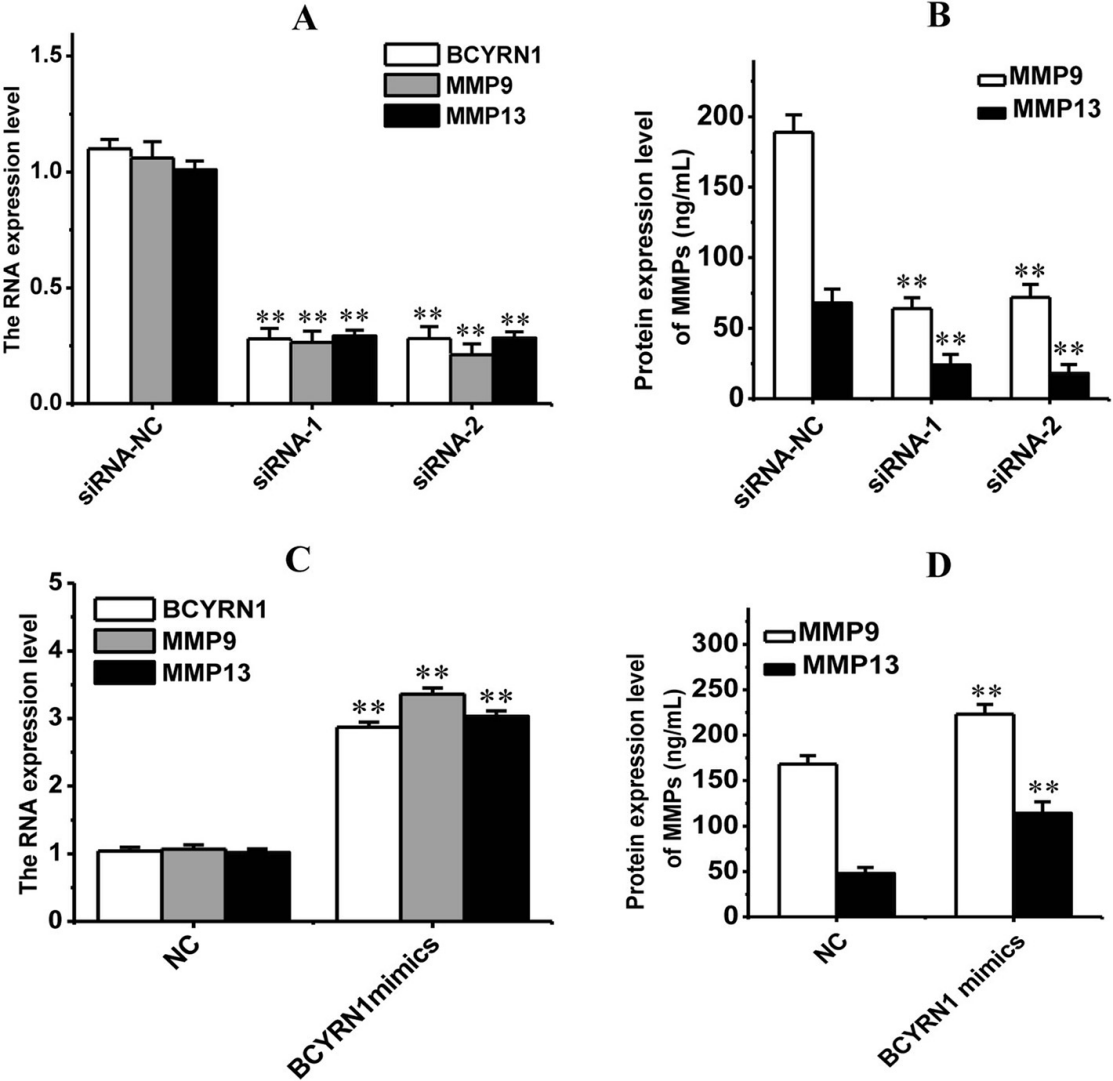
**The RNA (A, C) and protein (B, D) expression levels of MMP9 and MMP13 were both downregulated by BCYRN1 inhibition, and upregulated by BCYRN1 mimics.** siRNA-NC: siRNA negative control; siRNA-1: siRNA-BCYRN1-1; siRNA-2: siRNA-BCYRN1-2; NC: negative control for BCYRN1 mimics, only treated with Lipofectamine 2000. * *p* < 0.01, indicated a significant difference; ** *p* < 0.01, indicated a very significant difference, compared with negative control.

In the subsequent experiment, the RNA and protein levels of MMP9 and MMP13 in the A549 cells, which were respectively transfected with siRNA-*BCYRN1*-1 and siRNA-*BCYRN1*-2, were measured with real-time PCR and ELISA. As shown in Figure 4, the result demonstrated that both RNA and protein levels of MMP9 and MMP13 were significantly downregulated by the siRNAs against BCYRN1 and significantly upregulated by BCYRN1 mimics (compared with negative controls, **p < 0.01), which suggested a positive correlation between BCYRN1 and MMPs.

## Discussion

BCYRN1, also known as BC200, is generally not detected in normal tissue, in addition to the primate nervous system [11]. However, a low but detectable level of BCYRN1 was observed in non-tumor lung tissues in this study. It is very likely that the adjacent non-tumor tissues we obtained were so close to the tumor tissue that the adjacent tissues were not entirely separated from the tumor tissues, which may result in the detectable BCYRN1 level. Despite of this negligible imperfect, the main result agreed with previous reports [11,13]. High expressions of BCYRN1 in both NSCLC tissues and cell lines were confirmed by real-time PCR (Figure 1). The aberrant expression of cancer metastasis-associated lncRNA may drive tumorigenesis by regulating tumor suppressive and oncogenic pathways [21].

The c-MYC proto-oncogene is a frequently activated oncogene and is estimated to be involved in 20% of all human cancers, affecting many cancer deaths [22]. C-MYC has emerged foremost as a transcription factor, and is generally repressed in tumor suppressive pathways and activated in oncogenic ones, considerable parts of which are often metastasis-related [23,24]. In fact, it is suggested by recent estimates that c-MYC could regulate as many as 15% of genes in genomes from flies to humans, and its target genes have approached a total of more than 3000 human genes [22]. Based on what we’ve found online and the result confirmed by ChIP assay, we demonstrated a positive correlation between c-MYC and BCYRN1, indicating the former’s binding to the latter (Figure 2) in NSCLC cells. BCYRN1 is therefore a target gene of c-MYC. It is reported that some specific classes of genes that involve cell adhesion were affected by c-MYC, through which c-MYC plays a role in enhancing cell motility [22]. In our present study, the migration and invasion assays showed that BCYRN1 was indispensable in c-MYC-mediated metastasis of A549 cells (Figure 3). It was to say that, as a target gene of c-MYC, BCYRN1 mediated migration and invasion of A549 cells through a (some) pathway(s) that is still unknown.

In order to take a step into the mechanism of BCYRN1’s regulating tumor cell metastasis, the RNA and protein levels of representative MMPs, namely MMP9 and MMP13, were respectively measured. As a type of ECM-degrading enzymes, activation of MMPs has been proved by various studies to induce migration and invasion of tumor cells [25-27]. MMP9 is most likely involved in the initial degradation of the basement membrane surrounding the tumor, because MMP9 is induced under conditions that require tissue remodeling (including tumor invasion) [28,29]. And MMP13 has a broader substrate specificity than other collagenases and is able to cleave type IV, X and XIV collagens, tenascin, aggrecan core protein and fibronectin [30]. Therefore, the expressed MMP9 and MMP13 are able to regulate the metastasis of cancer cells [31]. In this study, real-time PCR and Western blotting analyses discovered that both RNA and protein levels of the MMP9 and MMP13 decreased with the reducing level of BCYRN1 and increased after treatment with BCYRN1 mimics (Figure 4). So, it was concluded that BCYRN1 somehow influenced the expressions of MMP9 and MMP13, thereby mediating cell migration and invasion in NSCLC.

In conclusion, cell metastasis of NSCLC was regulated by c-MYC-activated BCYRN1 through promoting the expressions of MMP9 and MMP13.
